# Development of a proxy-reported pulmonary outcome scale for preterm infants with bronchopulmonary dysplasia

**DOI:** 10.1186/1477-7525-9-55

**Published:** 2011-07-26

**Authors:** Sara E Massie, Sue Tolleson-Rinehart, Darren A DeWalt, Matthew M Laughon, Leslie M Powell, Wayne A Price

**Affiliations:** 1 Medicine Administration, School of Medicine, The University of North Carolina at Chapel Hill, Chapel Hill, NC, USA; 2 Department of Pediatrics, School of Medicine, The University of North Carolina at Chapel Hill, Chapel Hill, NC, USA; 3 North Carolina Translational and Clinical Sciences Institute, The University of North Carolina at Chapel Hill, Chapel Hill, NC, USA; 4 Cecil G. Sheps Center for Health Services Research and Division of General Medicine and Clinical Epidemiology, School of Medicine, The University of North Carolina at Chapel Hill, Chapel Hill, NC, USA

## Abstract

**Background:**

To develop an accurate, proxy-reported bedside measurement tool for assessment of the severity of bronchopulmonary dysplasia (also called chronic lung disease) in preterm infants to supplement providers' current biometric measurements of the disease.

**Methods:**

We adapted Patient-Reported Outcomes Measurement Information System (PROMIS) methodology to develop the Proxy-Reported Pulmonary Outcomes Scale (PRPOS). A multidisciplinary group of registered nurses, nurse practitioners, neonatologists, developmental specialists, and feeding specialists at five academic medical centers participated in the PRPOS development, which included five phases: (1) identification of domains, items, and responses; (2) item classification and selection using a modified Delphi process; (3) focus group exploration of items and response options; (4) cognitive interviews on a preliminary scale; and (5) final revision before field testing.

**Results:**

Each phase of the process helped us to identify, classify, review, and revise possible domains, questions, and response options. The final items for field testing include 26 questions or observations that a nurse assesses before, during, and after routine care time and feeding.

**Conclusions:**

We successfully created a prototype scale using modified PROMIS methodology. This process can serve as a model for the development of proxy-reported outcomes scales in other pediatric populations.

## Background

Bronchopulmonary dysplasia (BPD), or chronic lung disease (CLD), is one of the most common sequelae of preterm birth [1], and its severity is an important predictor of long-term outcomes in premature infants [2]. The infants most vulnerable to BPD are those born before the 28th week of gestation (extremely low gestational age newborns, ELGANs). Compared to their peers without lung disease, ELGANs with BPD have increased mortality [2,3]. Those who survive with BPD have prolonged initial hospitalizations [4] and an increased risk of neurodevelopmental impairment such as mental retardation and cerebral palsy [5-7]. These BPD-associated morbidities lead to increased family stress, economic hardship, and increased health care costs throughout childhood [4,8,9].

The most common definitions of BPD include the receipt of oxygen at 36 weeks post-menstrual age, with or without a physiologic test of oxygen dependency [10,11], and the National Institutes of Health (NIH) consensus categorization of "none," "mild," "moderate," and "severe," which is based on the duration of oxygen therapy and the amount of oxygen received at 36 weeks [12]. These NIH categories help determine the effect of therapies designed to reduce the incidence of BPD in a clinical trial, but they are not useful to providers who are attempting to examine the day-to-day pulmonary function of an infant, and this oxygen-based categorization does not capture the nuances of disease-related functional limitations.

A valid bedside assessment tool of pulmonary function will give clinicians and researchers a more effective way to test therapies by reliably identifying subtle effects on infant pulmonary function or by identifying subgroups of infants who respond to therapies such as diuretics or bronchodilators. Our goal was to develop a scale to assess the effects of lung disease on functional outcomes using proxy-reported measures. We adapted Patient-Reported Outcomes Measurement Information System (PROMIS) methodology, a widely recognized system of instrument item selection and refinement for patient-reported outcomes [13-18], to develop a parsimonious Proxy-Reported Pulmonary Outcomes Scale (PRPOS). Our most significant adaptation of current PROMIS methods is our entire reliance on proxy-reported measures for this neonatal population because of their inability to report on their own.

The ultimate goal of PRPOS is to provide clinicians with a set of items and responses in various functional domains that can discriminate between infants with differing degrees of BPD severity. Our secondary goal is to present a model instrument development process that might be replicated for use in diseases of infancy. This paper describes the first five of six steps in the scale development process: (1) identification of domains, items, and responses; (2) item classification and selection using a modified Delphi process; (3) focus group exploration of items and response options; (4) cognitive interviews of proxy reporters on a preliminary scale; (5) final revision before field testing; and (6) reliability testing (for which analysis is ongoing).

## Methods

**Figure 1:**
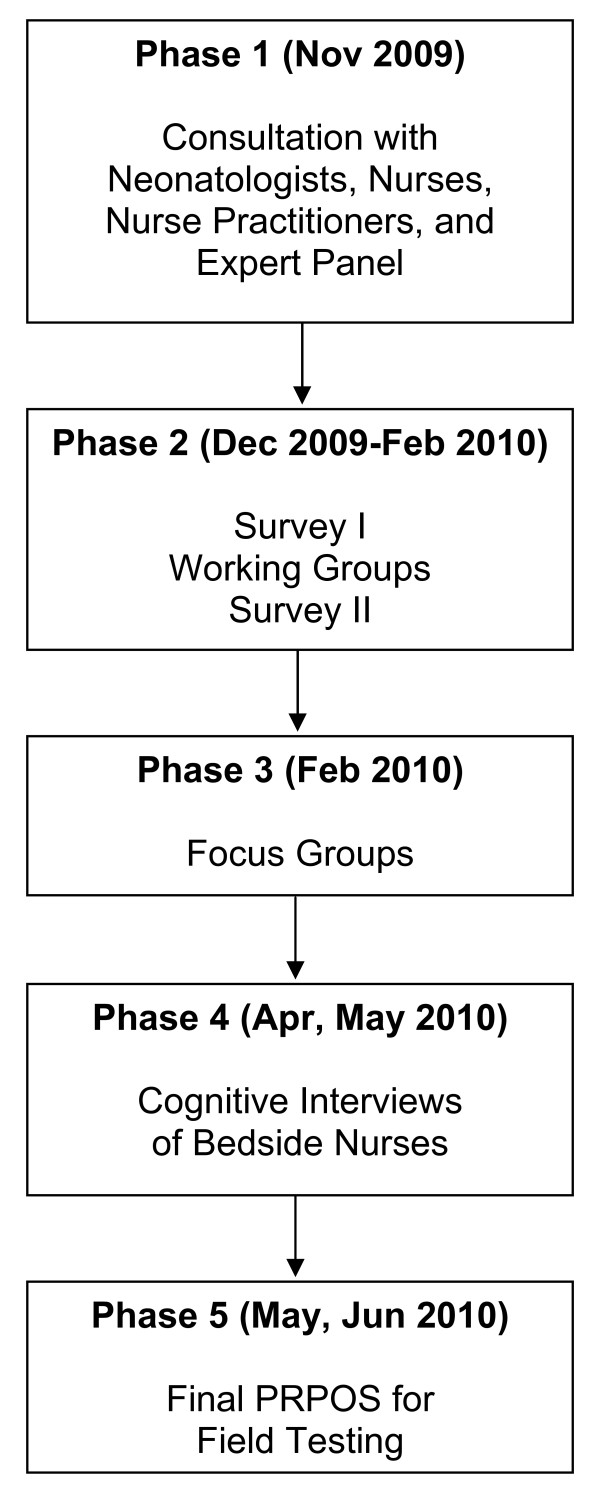
**PRPOS development phases**. Phases of development of the Proxy-Reported Pulmonary Outcomes Scale, from November 2009 to June 2010.

We developed PRPOS in the five phases illustrated in Figure 1.

### Phase 1: Identification of domains, items, and responses

We identified an appropriate set of activity domains and assessments for inclusion in the scale using face-to-face interviews with experienced neonatologists, nurses, and neonatal nurse practitioners at two academic medical centers (The University of North Carolina at Chapel Hill [UNC] and Duke University) and input from a panel of national experts in neonatology, pediatric pulmonology, feeding, and development.

We conducted interviews individually or in small groups using a "brainstorming" format. We asked respondents to use their clinical experience to identify characteristics of an infant diagnosed with BPD [CLD] at 36 weeks and any activities that precipitated these characteristics. During this phase of the process, items were included if at least two participants agreed on their discriminative utility, with the goal of identifying a complete set of potential items. The resulting set of activity domains and assessments, which grew in the course of the discussions from nine original "assessments and domains" to what began to be called 15 "qualities and conditions," was used in the next phase of the development process.

### Phase 2: Item classification and selection

We used a modified Delphi process, a method of obtaining consensus on a subject matter from experts in the field through anonymous solicitation or polling of their opinions [19], to identify, classify, review, and revise possible items and domains. Modified Delphi process participants included experienced neonatologists, nurses, and neonatal nurse practitioners, developmental specialists, and feeding specialists at five academic medical centers (UNC, Duke University, Stanford University, University of Alabama at Birmingham [UAB], and University of Iowa [Iowa]).

Our modified Delphi process included three steps: (1) a survey, (2) working group meetings, and (3) a second survey reflecting areas where consensus had not yet been achieved. The surveys were designed and administered using the web-based survey software Qualtrics (Provo, UT), and each respondent received a unique URL to the surveys. The entire process took place from December 2009 to February 2010.

We invited 59 clinicians from five academic medical centers to participate in the two surveys (Table 1); in addition, we asked our eight expert panel members to take the second survey.

**Table 1:** Demographic information on participants in the modified Delphi process

	Survey 1	Working Groups	Survey 2
**Total No. Participants**	38	14	43

Missing data	3		1

**Institution, n (%)**			

UNC	13 (34%)	7 (50%)	9 (21%)

Duke	6 (16%)	7 (50%)	7 (16%)

Stanford	7 (18%)	0	9 (21%)

UAB	1 (3%)	0	3 (7%)

Iowa	8 (21%)	0	8 (19%)

Expert Panel	0	0	7 (16%)

**Role, n (%)**			

MD	10 (26%)	2 (14.3%)	14 (33%)

NP	9 (24%)	1 (7.1%)	10 (23%)

RN	10 (26%)	6 (42.9%)	13 (30%)

Specialist	6 (16%)	5 (35.7%)	6 (14%)

Years in Practice, mean*			

MD	14.7	12.3	11.4

NP	21.1	30	23.5

RN	18.8	15	20.1

Specialist	18.7	17.5	15.3

*Note: Years in practice have missing data for four cases in survey 1 and 16 cases in survey 2.

The first survey (step one) had three parts. In part one, respondents described how certain qualities or conditions (alertness, tone of back/trunk, lower body, and upper body, eye appearance, eyebrow appearance, desaturations, presence of tachypnea, recovery time from tachypnea, retractions, and heart rate) appear in infants with four levels of BPD [CLD] severity--none, mild, moderate, severe--in three situations (e.g., at baseline before care, during care time, and during the first five minutes of feeding). Table 2 presents the scenarios used to describe level of CLD severity. Respondents also described the appearance of three feeding cues: opening the mouth, dropping the tongue, and the position of the chin. The survey provided three "other" categories where respondents could fill in additional characteristics they thought were important and describe the appearance of those characteristics in infants at each of the disease states.

**Table 2:** Scenarios to describe level of CLD severity

Severity Level	Scenarios
No CLD	Baby Doe was extubated to CPAP and off supplemental oxygen by DOL^a ^22. He is now DOL 84 (36 weeks corrected age). Baby Doe has NO CLD.

Mild CLD	Baby Doe came off all oxygen on DOL 65. He is now DOL 84 (36 weeks corrected age). Baby Doe has MILD CLD.

Moderate CLD	Baby Doe is now DOL 84 (36 weeks corrected age) and on 0.1 lpm oxygen. Baby Doe has MODERATE CLD.

Severe CLD	Baby Doe is now DOL 84 (36 weeks corrected age) and on high-flow oxygen blended to an FIO2 of 0.65. Baby Doe has SEVERE CLD.

^a^DOL - day of life.

In part two of the survey, respondents rated how well each of the observation domains and feeding cues would discriminate levels of CLD severity using a scale of 1 to 9, where 1 = not at all well and 9 = extremely well.

In part three, respondents provided open-ended feedback on the types of things that should be recorded before the assessment (e.g., whether a retinopathy of prematurity exam had taken place that day, or the timing of a furosemide dose) and made comments on other things we should consider in developing the scale.

Following the survey, we conducted three multidisciplinary workgroups (step two of the modified Delphi process) at UNC and Duke. At the start of the workgroups, we asked participants to score how well a set of items--quality of sleep; alertness, arousability, facial expression; disorganization; difficulty in calming; color change; tone; and feeding mechanics---reflects the severity of CLD in an infant during five states (sleep, transition, awake state, care time, and feeding) using a five point scale (0 = no; 1 = some; 2 = moderately, 3 = pretty closely; and 4 = yes, very much). We then had guided discussions in which we asked participants to help refine our set of domains, narrow similar terms to a single, best descriptor, and clarify and simplify complex items. At the end of the workgroup, participants completed the score card again, and we determined whether discussion had changed preferences.

The feedback we received from the working groups contributed to development of our second survey (step 3), in which respondents estimated at what severity of lung disease they might observe a particular behavior or action and how well those items discriminate levels of CLD severity. Table 3 lists the five behavior domains. We also asked whether the following terms were familiar and useful in describing breathing: intercostal, subcostal, and substernal retractions; head bobbing; and nasal flaring. The survey included space for respondents to provide additional comments. At the conclusion of the modified Delphi process, we developed a preliminary scale.

**Table 3:** Domains and behaviors used in survey 2

Domain	Behavior
Sleep	Interrupted sleep/restlessness
	
	Excessive sleepiness
	
	Sustained active or quiet sleep

Arousal/transition	Transitions well between states
	
	Arouses easily, but to agitation
	
	Arouses with difficulty

Awake state: General state during care time	Mainly quiet alert or active alert
	
	Wiped out, persistent drowsiness
	
	Restless, agitated

Awake state: Calming during care time	Calms, but with some difficulty
	
	Irritable, not easily calmed
	
	Calms with containment, voice soothing

Awake state: Eye appearance during care time	Eyes intermittently opened and closed
	
	Eyes tightly closed
	
	Engaged/alert
	
	Panicked/wide-eyed
	
	Glazed/blank

Awake state: Eyebrow appearance during care time	Raised
	
	Relaxed/neutral
	
	Furrowed

Awake state: Color change during care time	Mottled
	
	Pale
	
	Dusky
	
	None

Awake state: Tone during care time	Arched/shoulders elevated or retracted
	
	Floppy
	
	Mainly flexed/hands loosely flexed or opened and closed
	
	Some increased extensor tone, fingers splayed

Feeding mechanics: Rooting/feeding cues	Roots and initiates feeding cues independently
	
	Minimal cues/rooting

Feeding mechanics: Mouth/tongue position during first 5 minutes of feeding	Opened and rounded/seals on nipple spontaneously or with prompting
	
	Turns head away/hesitant to open mouth
	
	Refuses to eat
	
	Open mouth posture/tongue and chin positioned to open airway

Feeding mechanics: Tone during first 5 minutes of feeding	Floppy
	
	Mainly flexed/hands loosely flexed or opened and closed
	
	Arched/shoulders elevated or retracted
	
	Some increased extensor tone, fingers splayed

Feeding mechanics: Desaturation during first 5 minutes of feeding	Not able to accept nipple without desats
	
	Frequent breaks required for pacing
	
	Desats with sustained sucking; recovers with intervention

Feeding mechanics: Respiratory rate (RR) with feeding	RR above baseline during sucking pause periods/recovers slowly
	
	Tachypnea at onset of feeding only
	
	RR above baseline during sucking pause periods/recovers quickly

Respiratory: desaturation during care time	Severe or frequent
	
	Mild or intermittent or occasional
	
	Moderate or somewhat common

Respiratory: tachypnea during care time	Constant
	
	No tachypnea
	
	Occasional or intermittent

### Phase 3: Focus groups

In February 2010, we conducted two focus groups of bedside nurses, a physical therapist, and a developmental specialist to clarify domains, confirm item definitions, and refine the wording of potential scale items and corresponding response options [13,20]. An experienced focus group moderator conducted both focus groups, and members of the research team observed the discussions and provided background and clarification when necessary. The moderator used a semi-structured interview guide to elicit group participation and discussion on specific topic areas. We audiorecorded the focus group sessions and compared and collated notes taken by investigators in the group with the moderator's notes from the transcripts.

Each focus group was presented with the same scenario describing the clinical course of a premature infant at 36 weeks, and then asked to think about the infant in four disease states, no CLD, mild, moderate and severe CLD (see Additional File 1, Box S1). The focus group moderator instructed the participants to refer to the scenario throughout the discussion. Questions during the discussion centered on nine areas (Table 4).

**Table 4:** Sample focus group questions from nine domains

Topic area	Sample questions
Arousal from sleep	How would you describe babies who 'arouse with difficulty'? What would that look like?

Calming	What would "may have trouble calming" look like if you were describing a baby with moderate CLD? What would someone observe? How about with severe CLD?

Agitation	How would you describe a CLD baby who is 'very agitated'? What are all the observations you might make about a baby at the far end of that spectrum (severe disease)?

Energy level/activity	Describe a CLD baby in "a high energy" state. How, if at all, would an agitated baby look different from a baby in a state of high energy level/activity level?

Eye appearance	Is it helpful to include a 'glazed/blank' assessment of eye appearance? If so, is 'glazed/blank' on the spectrum from 'engaged' to 'panicked/wide-eyed' or is 'glazed/blank' indicating something different?

Color change	What color change do you observe in babies with CLD? What words best describe that color change?

Tone	What is a specific word or a modifier that describes a baby that has such bad lung disease and is so tired and wiped out that they become low-tone?

Desaturations	Do babies with no lung disease sometimes desat? Would 'normal' include an occasional desat?

Respiratory rate	How would you describe respiratory rate with feeding in a baby with no CLD?

### Phase 4: Cognitive interviews

Following the focus groups, we conducted semi-structured cognitive interviews to obtain information about what items actually meant to potential respondents in terms of their comprehension of individual questions (i.e., the question intent and meaning of terms), the sense of the questions overall, retrieval from memory of relevant information (i.e., recallability of information and recall strategy), decision processes, response processes, and instructions for using the tool [13,18,21,22].

The cognitive interviews were approved by the Institutional Review Board at UNC, and all interviewees gave their informed consent prior to the interview. The interviews took place in April and May 2010 and included bedside nurses from three academic medical centers (UNC, Stanford, and Iowa), chosen to elucidate possible regional differences in response to terms. In our cognitive interview process, a bedside nurse used the scale on an infant and then participated in a cognitive interview. The experienced cognitive interviewer followed a semi-structured interview guide with questions about each item, the overall scale, and the directions.

Examples of the cognitive interview questions include

• On a scale of 1 to 5, with 1 being easiest and 5 being hardest, how easy or hard was it to choose an answer?

• How sure are you of your answer? *-or- *How sure are you that it is [X]?

• Would it be easier for you if you could choose from fewer options? (*If yes, probe: *what response options would you eliminate?)

• Would it be easier for you if you could choose from more options? (*If yes, probe: *what other response options would you like to see here?)

• Is there another response that should be added that would more fully describe what you observe?

• Why do you say [X]? -*or- *Tell me why you chose [answer] instead of some other answer on the list.

After the first three interviews, we assessed each nurse's feedback and revised items and response options in the scale that respondents had thought were unclear. We then conducted three more interviews and made minor changes to the scale after each one.

### Phase 5: Final scale revision

We used the results of the focus groups and cognitive interviews to develop a prototype PRPOS and prepare it for field testing in five geographically dispersed academic centers with varying rates of BPD.

## Results

### Phase 1: Identification of domains, items, and responses

During the brainstorming phase, 15 experienced clinicians identified an initial item pool of nine activity domains and nine assessments (Table 5). The national expert panel included two neonatologists, two pediatric pulmonologists, two infant feeding experts, and two neurodevelopmental specialists (seven from the United States and one from Canada). They confirmed that these domains and assessments were comprehensive, observable, and related to CLD at age 36 weeks adjusted gestational age. However, the expert panel raised a potential concern about assessing feeding behaviors because of the interaction of immaturity, respiratory disease, and feeder skill. Based on this input, we modified the feeding assessment to include only the initial period of feeding.

**Table 5:** Initial set of activity domains and assessments

Activity Domains	Assessments
At rest	Position: Tone (arched, relaxed)

Feeding by mouth	Pulse oximetry: Desaturation (length, depth)

Oro-gastric feeding	Retraction (subcostal, intercostal, head bob)

Handling/transitions/care time	Tachypnea (change in respiratory rate, time to baseline)

Family holding	Apnea (number, severity)

Noise	Heart rate (bradycardia)

Transition to awake	Alertness (engages, averts gaze, frantic)

Stooling	Circumoral cyanosis (presence of)

Sleep time (quiet alert/engaged periods versus prolonged sleep time)	Oro-motor dysfunction

Using input from the face-to-face interviews and expert panel, we arrived at a set of 15 activity domains and assessments, or "qualities and conditions," to be included in the next phase of the development process.

### Phase 2: Item classification and selection (modified Delphi and workgroups)

We received 38 responses to the first survey (response rate = 64%) and 43 responses to the second survey (response rate = 64%). Seventeen people took part in the working groups: ten from UNC, including nurses and a feeding specialist, and seven from Duke, including developmental/family specialists, researchers, and a nurse.

#### First Survey

The open-ended responses to the first survey provided us with user-generated, specific terms and phrases with which respondents could describe an infant's appearance at the four levels of BPD severity. Nurses and neonatal nurse practitioners provided more detailed descriptions than did neonatologists, and the feeding and developmental specialists provided more nuanced responses about feeding and development.

Table 6 shows that, on average, registered nurses, nurse practitioners, neonatologists, and developmental and feeding specialists scored alertness, tone, eyes, eyebrows, and feeding cues mid-range (4-6) on the scale. Desaturation, tachypnea over baseline, time to recover from tachypnea, retractions received high scores (8 or 9). Nurses and specialists were more likely than were physicians to rate aspects of tone and feeding as valuable discriminators of levels of CLD severity.

**Table 6:** Survey 1 results of average ratings of appropriateness of CLD observation

Observation domain	MDs (n = 10)	RNs/NPs (n = 19)	Specialists (n = 6)
Alertness, mean (SD)	4 (2.03)	5 (2.29)	5 (2.48)

Tone:			

back/trunk	4 (2.12)	5 (2.03)	6 (2.77)

upper body	3 (1.77)	6 (2.02)*	6 (2.34)*

lower body	3 (1.81)	5 (1.76)*	4 (2.07)

Eyes	4 (2.20)	6 (1.97)	6 (2.51)

Eyebrows	4 (2.10)	6 (2.06)	6 (2.25)

Feeding cues:			

opens mouth	4 (1.98)	7 (1.46)*	6 (2.86)*

drops tongue	4 (1.81)	7 (1.73)*	6 (2.83)

position	5 (2.20)	7 (1.83)	6 (2.93)

Desaturation	8 (1.90)	8 (1.00)	8 (0.84)

Tachypnea:			

over baseline	8 (1.57)	8 (0.94)	9 (0.55)

time to recover	8 (1.51)	8 (0.61)	9 (0.55)

Retractions	8 (1.81)	8 (0.97)	9 (0.55)

Heart rate	6 (1.72)	7 (1.09)	7 (1.50)

*p < 0.05 vs MD responses (ANOVA with post-hoc analysis using the Student-Newman-Keuls all pairwise multiple comparison procedure)

Respondents reported that pre-assessment data should include information on the clinical environment (e.g., parent visits, room noise), administration and timing of medications (e.g., timing of last steroid course, dose of caffeine/aminophylline), procedures and tests (e.g., laboratory tests, immunizations, radiology visit), and respiratory support (e.g., type and magnitude of support).

#### Workgroup Feedback

The workgroup participants assisted in narrowing multiple terms to a single, best term for 12 items. For example, eyebrow descriptors "furrowed," "scrunched," "contracted," and "tense" were narrowed to "furrowed." In addition, participants clarified, defined, or distinguished similar descriptions for eight items. For instance, participants helped discriminate between eyes closed due to stress, described by the term "eyes tightly closed," and eye closure that does not indicate distress, denoted by "closed and sleepy" eyes. In three cases, workgroup participants simplified terms; for example, we reduced descriptions of musculoskeletal tone from four to three because of clinicians' inability to discriminate accurately between four different levels.

Participants also highlighted areas of uncertainty, expressing concern that some of our feeding items (mouth/tongue position; rooting/feeding cues) might be influenced by the feeder's technique and level of experience or the infant's development and feeding skills, rather than by the infant's level of CLD severity. The groups also noted that it is difficult to decipher whether "raised" and "furrowed" eyebrows signal distress related to the infant's CLD.

When we asked workgroup members to rescore after discussion, their responses did not change significantly from what they reported before discussion. Overall, most items scored as "moderately" or "pretty closely" reflecting severity of CLD in infants.

#### Second Survey

Results from the second survey of the modified Delphi process suggested that we had a range of behaviors and actions that would indicate different levels of CLD severity for each domain (see Additional File 2, Table S1). For five of the domains (tone and desaturations during the first five minutes of feeding, respiratory rate with feeding, and calming and desaturations during care time), we did not have a descriptive behavior or action that would reflect the absence of disease, or "no CLD". Thus, we added a descriptor that reflected no CLD more clearly. For five domains (sleep, arousal/transition, general state during care time, color change, and feeding cues), we had descriptive behaviors or actions that showed overlap between moderate and severe disease. Most respondents (81%) reported that intercostal, subcostal, and substernal retractions, head bobbing, and nasal flaring were familiar and/or useful terms to describe breathing. A few respondents (16%) noted other degrees to consider between "barely visible" and "pronounced," and a few others (9%) did not find the term "head bob" familiar or useful.

We chose eleven areas for further discussion, expansion, and clarification using focus groups. We eliminated four potential assessment domains (sleep, rooting/feeding cues, mouth/tongue position, and tone during first five minutes of feeding) because of difficulty in defining an appropriate scale (sleep) or low scores on the CLD discrimination question. We also added two areas--retractions and nasal flaring-- for inclusion on the tool, but we determined that we did not need to explore these further during the focus groups.

### Phase 3: Focus Groups

Eighteen beside nurses and specialists participated in the two focus groups, with nine participants in each group. All participants had at least three years of experience in the neonatal intensive care unit. The focus group discussions helped us to confirm response options for our items and determine the scale endpoints from no disease to severe CLD. Focus groups also helped us discover which terms should not be used as response options (e.g., "mottled" to describe the infant's color, and "floppy" or "hypotonic" to describe the infant's tone). As we note above, we began by presenting the focus groups with eleven areas, arousal, general state during care time, calming, eyes, eyebrows, color, tone, desaturations during feeding, respiratory rate during feeding, desaturations, and tachypnea, and asked group members to discuss transition/arousal, calming, agitation and energy/activity level, eye appearance, color change, tone, desaturations, and respiratory rate. We also asked focus group members to think about descriptors of general state--mainly calm or quiet, restless, agitated or irritable, distressed, and frantic--and of the ability to calm--self-calms, calms with containment, voice soothing, irritable, not easily calmed, frantic/inconsolable. In the course of listening to focus group discussion, we chose to eliminate the questions about color and tone, and also to eliminate questions about eyebrows, but retain questions on eyes, and add questions about respiratory rate and desaturation during both care time and feeding.

### Phase 4: Cognitive Interviews

Six bedside nurses from three academic medical centers, UNC (n = 3), Stanford University (n = 2), and the University of Iowa (n = 1) participated in one-hour cognitive interviews.

Overall, the nurses reported that the questions were easy to answer. Interview respondents found that the tool's instructions were understandable for the overall assessment and the care time portion of it, but they found the instructions less clear for the feeding portion of the assessment. At least one respondent suggested wording changes to the response options of 12 of 20 questions, but half or more of the respondents suggested changes to the response options for only these four questions: (1) How would you describe the infant's general state?; (2) How would you describe the infant's tone?; (3) How do the infant's eyes appear as you begin care?; and (4) How would you describe the infant's endurance during care time?

In response to these cognitive interview results, we changed the response options in four cases about which at least half the respondents had suggestions. The old and new responses to the questions are presented in Table 7. To illustrate the evolving refinement of responses, we initially included two additional response options to the general state question: "sleeping" and "tired." After testing this twice, we realized that the question should actually be divided into two questions--one on "general state" and one on "general status."

**Table 7:** Response option rewording after cognitive interviews

Question	Original Response Options	Revised Response Options
How would you describe the infant's general state?	Mainly calm or quiet	Active or quiet sleep
	
	Restless	Drowsy - eyes open and closed
	
	Agitated or irritable	Awake
	
	Distressed	
	
	Frantic	

How would you describe the infant's general status?*	n/a	Mainly calm or quiet
	
	n/a	Tired
	
	n/a	Restless
	
	n/a	Agitated or irritable
	
	n/a	Distressed
	
	n/a	Frantic

How would you describe the infant's tone?	Soft flexion	Soft or neutral flexion
	
	Some increased extensor tone, fingers splayed	Arms extended
	
	Increased extensor tone with arching and/or shoulders elevated or retracted	Arms extended with arching and/or shoulders elevated or retracted
	
		Limp (wiped out)

How do the infant's eyes appear?	Asleep - can't observe	Asleep or closed - can't observe
	
	Engaged/alert/bright-eyed	Crying
	
	Easily distracted	Tired
	
	Panicked/wide-eyed	Engaged or alert
	
		Easily distracted
	
		Panicked

How would you describe the infant's endurance during care time? ("Endurance" revised to "stamina")	No fatigue (tolerates care time well	Sufficient stamina - tolerated care time well
	
	Minimal fatigue (shows some signs of fatigue with care but recovers quickly)	Tired some with care but recovered quickly
	
	Moderate fatigue (frequent signs of fatigue with care but recovers with pause)	Tired easily with care but recovered with pause
	
	Easily fatigued ('wiped out' 3-5 minutes into normal care time)	Tired easily without recovery ('wiped out' 3-5 minutes into normal care time)

* new question broken out of "general state" question as a result of discussion, thus, original response not applicable (n/a)

### Phase 5: Final item revision

We refined the directions for using the scale, particularly for the feeding assessment section. We defined "desaturation" as an oxygen saturation of less than 80%, and we defined "increased respiratory rate" as a respiratory rate above 60 or, if the infant's baseline respiratory rate was already above 60, an "increase" is defined as a respiratory rate above the baseline. We provided instructions for how to calculate the baseline respiratory rate--count for 30 seconds, then multiply by 2--and we revised other question wording and response options, examples of which can be seen in Table 8.

**Table 8:** Examples of question and response option wording changes to the PRPOS

Original	Revision
*Question: *Does this infant's care plan or orders require or allow an increase in oxygen support during care time? *Response options: *No, Yes	Split "yes" response option into "yes - required" and "yes - allowed"

*Question: *How would you describe the infant's general state? *Response options: *Asleep, Drowsy - eyes open and closed, Awake	Changed "asleep" response option to "asleep (active sleep or quiet sleep)"

*Question: *How would you describe the infant's color?	Added instruction to ignore jaundice.

*Question: *How would you describe the infant's breathing?	Reworded question to "How would you describe the greatest degree of retractions you observe?"

*Question: *How would you describe the infant's tone? *Response options: *Soft flexion; some increased extensor tone, fingers splayed; increased extensor tone with arching and/or shoulders elevated and retracted	Revised response options to "soft or neutral flexion," "arms extended," "arms extended with arching and/or shoulders elevated or retracted," lip (wiped out)

*Question: *How do the infant's eyes appear as you begin care? *Response options: *asleep- can't observe, engaged/alert/bright-eyed, easily distracted, panicked/wide-eyed	Revised response options to "asleep or closed - can't observe," "crying," "tired," "engaged or alert," "easily distracted," and "panicked"

## Discussion

The use of the PROMIS methodology in PRPOS's development assures us that the creation of the instrument has been both transparent and replicable expert clinical judgment from registered nurses, neonatal nurse practitioners, neonatologists, and developmental and feeding specialists has informed all the phases of the development process. We continually refined the scale's potential set of items and response options with the goal of achieving a parsimonious set of items going into the cognitive interviews. We did not have to remove any items during the final scale revision. The prototype scale includes 26 questions about the infant that a nurse assesses before, during, and after a routine care time and feeding, and takes less than 2 minutes to complete.

Our scale development process was similar to, but more broadly inclusive and iterative than, the development of the Premature Infant Pain Profile [23,24] because of our use of modified Delphi surveys, workgroups, focus groups, and cognitive interviews. We used the more extensive and rigorous modified PROMIS methodology in an attempt to overcome some of the inherent limitations of proxy measures and to accomplish much of the work of establishing valid and reliable items prospectively, rather than depending entirely on retrospective testing of measures. Each phase of the development process produced uniquely valuable information. The initial consultation with expert providers helped us explore and define the domains we needed to measure. The modified Delphi Process, including the two surveys interrupted by workgroup discussion, gave us enormous insight into shared--and unshared--conceptual underpinnings to common terms. The focus groups of end-users--the bedside neonatal intensive care unit nurses who care for infants with BPD--reassured us that we had succeeded in narrowing the domains to the minimum number that adequately describes BPD infants' disease state, to decrease the burden of administration. Finally, the cognitive interviewing gave us an exceptional opportunity to query users' experience with the instrument itself: "Was it understandable? Easy to complete? Effective? Did response categories mean to users what we intended them to mean?" We expect that completion of all these steps will enhance the usefulness of each individual item and enhance the usability of these assessment items across different clinical settings.

Each instrument development phase could not alone lead to a successful product, but no phase was dispensable, and, taken together, they have generated a set of items ready for quantitative assessment. Our development process is limited by the fact that it is performed only in academic medical centers, although it is reasonable to assume that most non-academic center neonatal intensive care units would share many features of the academic medical center environment. Our focus groups were conducted at only two neonatal intensive care units both located in a single state, opening the possibility of limitations by region, or practice culture. Our more geographically dispersed cognitive interviewing and field testing should help us identify any such problems.

The PRPOS is currently undergoing field testing at five academic medical centers, where bedside nurses are applying the assessment tool to a cohort of 150-200 neonates (25-40 per institution) between 23 and 30-6 weeks gestational age at birth (excluding infants with chromosomal abnormalities) and between 36-0 and 36-6 weeks postmenstrual age. At the conclusion of field testing, we will perform psychometric analyses of the data to test item validity and reliability, for the purpose of further scale refinement.

## Conclusions

We expect that use of the PRPOS to assess observable, functional domains will greatly enhance the current unidimensional assessment of BPD severity based on oxygen use alone. For example, the PRPOS might allow clinicians and researchers to test therapies for BPD more effectively by accurately identifying subtle effects on lung function. In addition, refinement in the definition of BPD may allow more accurate prediction of important outcomes such as hospital length of stay and re-hospitalization after discharge, and further refine the relationship between BPD and neurodevelopmental outcome.

Use of a structured approach modelled on the rigorous PROMIS methodology helped us develop and refine a proxy-reported measurement instrument over a short period of time, while maintaining precision, clarity, discrimination, and comprehensiveness balanced with parsimony. This approach will serve as a useful model for others interested in developing proxy-reported outcomes measures.

## List of Abbreviations

BPD: bronchopulmonary dysplasia; CLD: chronic lung disease; ELGAN: extremely low gestational age newborn; PRPOS: proxy: reported pulmonary outcome scale.

## Competing interests

The authors declare that they have no competing interests.

## Authors' contributions

Research question: WAP, MML; Study conceptualization and design: WAP, MML, STR, DAD, SEM; Data collection: WAP, SEM, LMP; Data analysis and interpretation: WAP, SEM, STR, DAD; Initial draft and revisions of manuscript: SEM, WAP, STR; Manuscript revision: DAD, MML, LMP. All authors read and approved the final manuscript.

## Supplementary Material

Additional file 1 **Box S1. Focus Group Scenario**. This file presents the scenario used in the focus group discussions. Click here for file

Additional file 2 **Table S1. Survey 2 results for CLD severity classification of behaviors and actions in each domain**. This file shows a table of the domains and behaviors/actions used in the second survey, with an indication of whether the behavior/action was classified as being characteristic of no, mild, moderate, or severe lung disease. Click here for file
