# Supplementary material for: Development of a proxy-reported pulmonary outcome scale for preterm infants with bronchopulmonary dysplasia
Source: Health Qual Life Outcomes. 2011 Jul 26;9:55. doi: 10.1186/1477-7525-9-55 (PMC3161834; doi:10.1186/1477-7525-9-55)
Supplement: Additional file 1 — Box S1. Focus Group Scenario. This file presents the scenario used in the focus group discussions. [file 1477-7525-9-55-S1.DOC]

**Focus Group Scenario**

At the beginning of each focus group, participants were given this scenario: “*Baby Jane Doe was born at 24 weeks gestation, placed on a ventilator for 21 days after receiving surfactant, then extubated to CPAP. Her head ultrasound shows no IVH (bleeding in the brain) and her ROP exams have been normal. She is now DOL 84 (36 weeks corrected age). (This is a baby who has no medical issues other than CLD).*” Participants were then asked to think about the infant in four disease states:

- no CLD (she is on room air),
- mild CLD (she was on CPAP until DOL 28, then nasal cannula oxygen until DOL 75 but is now on room air),
- moderate CLD (she was on CPAP until DOL 35 [25-35% oxygen] and is now on 0.1 lpm nasal cannula), and
- severe CLD (she was on CPAP until DOL 50 [50-60% oxygen] and is now on high-flow oxygen at 65%).
