# Supplementary material for: Development of a proxy-reported pulmonary outcome scale for preterm infants with bronchopulmonary dysplasia
Source: Health Qual Life Outcomes. 2011 Jul 26;9:55. doi: 10.1186/1477-7525-9-55 (PMC3161834; doi:10.1186/1477-7525-9-55)
Supplement: Additional file 2 — Table S1. Survey 2 results for CLD severity classification of behaviors and actions in each domain. This file shows a table of the domains and behaviors/actions used in the second survey, with an indication of whether the behavior/action was classified as being characteristic of no, mild, moderate, or severe lung disease. [file 1477-7525-9-55-S2.DOC]

**Additional file 2: Table S1. Survey 2 results for CLD severity classification of behaviou**rs and actions in each domain

| **Domain** | **Behaviour or Action** | **No** | **Mild** | **Moderate** | **Severe** |
| --- | --- | --- | --- | --- | --- |
| Sleep | Sustained active or quiet | x | x |  |  |
|  | Interrupted sleep/restlessness |  |  | x | x |
|  | Excessive sleepiness |  |  | x | x |
| Arousal/ transition | Transitions well | x | x |  |  |
|  | Arouses easily, but to agitation |  |  | x | x |
|  | Arouses with difficulty |  |  | x | x |
| General state during care time | Mainly quiet or active alert | x | x |  |  |
|  | Restless, agitated |  |  | x | x |
|  | Wiped out, persistent drowsiness |  |  | x | x |
| Calming during care time | Calms with containment, voice soothing |  | x |  |  |
|  | Calms, but with some difficulty |  |  | x |  |
|  | Irritable, but not easily calmed |  |  | x | x |
| Eye appearance | Engaged/alert | x | x |  |  |
|  | Glazed/blank |  |  | x | x |
|  | Intermittent open/closed |  | x |  |  |
|  | Tightly closed |  |  | x | x |
|  | Panicked/wide-eyed |  |  |  | x |
| Eyebrow appearance | Relaxed/neutral | x | x |  |  |
|  | Raised |  |  | x |  |
|  | Furrowed |  |  | x | x |
| Colour change | None | x | x |  |  |
|  | Pale |  | x | x | x |
|  | Dusky |  |  | x | x |
|  | Mottled |  |  | x | x |
| Tone | Mainly flexed/hands loosely flexed or open/closed | x | x |  |  |
|  | Some increased extensor tone, fingers splayed |  |  | x |  |
|  | Arched/shoulders elevated or retracted |  |  | x | x |
|  | Floppy |  |  |  | x |
| Rooting/ feeding cues | Roots and initiates feeding cues independently | x | x |  |  |
|  | Minimal cues/rooting |  |  | x | x |
| Mouth/ tongue position | Open and rounded / seals on nipple spontaneously | x | x |  |  |
|  | Needs assistance to maintain seal |  |  | x | x |
|  | Turns head away/hesitant to open mouth |  |  | x | x |
|  | Open mouth/tongue, chin positioned to open airway |  | x | x |  |
|  | Refuses to eat |  |  | x | x |
| Tone during first 5 minutes of feeding | Mainly flexed/hands loosely flexed or open/closed |  | x |  |  |
|  | Some increased extensor tone, fingers splayed |  |  | x |  |
|  | Arched/shoulders elevated or retracted |  |  | x | x |
|  | Floppy |  |  |  | x |
| Desats during first 5 minutes | Desats with sustained sucking, recovers with intervention |  |  | x |  |
|  | Frequent breaks required for pacing |  |  | x | x |
|  | Not able to accept nipple without desats |  |  |  | x |
| Respiratory rate with feeding | Tachypnea at onset of feeding only |  | x | x |  |
|  | RR above baseline/recovers quickly |  | x | x |  |
|  | RR above baseline/recovers slowly |  |  | x | x |
| Desats during care time | Desats mild or intermittent or occasional |  | x |  |  |
|  | Desats moderate or somewhat common |  |  | x |  |
|  | Desats severe or frequent |  |  |  | x |
| Tachypnea during care time | No tachypnea | x |  |  |  |
|  | Occasional or intermittent tachypnea |  | x | x |  |
|  | Constant tachypnea |  |  |  | x |
